# Physical, cognitive, and psychosocial fatigue are differently related to cortical complexity of superior temporal and frontal brain regions in Crohn’s disease

**DOI:** 10.3389/fnimg.2026.1814006

**Published:** 2026-04-16

**Authors:** Theresa A. McIver, Charles N. Bernstein, Ruth Ann Marrie, John D. Fisk, Chase R. Figley, Jennifer Kornelsen

**Affiliations:** 1 Department of Medicine, University of Manitoba, Winnipeg, MB, Canada; 2 IBD Clinical and Research Centre, University of Manitoba, Winnipeg, MB, Canada; 3 Department of Medicine, Dalhousie University, Halifax, NSW, Canada; 4 Departments of Psychiatry, Medicine, Psychology, & Neuroscience, Dalhousie University, Halifax, NSW, Canada; 5 Department of Radiology, University of Manitoba, Winnipeg, MB, Canada

**Keywords:** brain-gut axis, cortical complexity, Crohn’s disease, fatigue, inflammatory bowel disease, MRI, disease activity

## Abstract

**Introduction:**

Fatigue is common in persons with Crohn’s disease, negatively impacting quality of life in both active and remitted disease state. Neural correlates of fatigue in Crohn’s disease are understudied, particularly relative to the separate impacts of physical, cognitive, and psychosocial fatigue. The potential moderating role of cortical complexity on the relationship between disease activity and fatigue has yet to be examined.

**Methods:**

Forty-nine participants with Crohn’s disease and 49 healthy control participants completed the Fatigue Impact Scale (which includes physical, cognitive, and psychosocial subscales) and whole-brain T1-weighted magnetic resonance imaging. Cortical complexity analyses were performed in CAT12, including within- and between-group analyses.

**Results:**

In the Crohn’s disease group, greater fatigue across all domains was associated with lower cortical complexity in the right superior temporal gyrus. Physical and cognitive impacts of fatigue were differently related to cortical complexity in the superior frontal and supramarginal gyri. Cortical complexity in the healthy control group was exclusively, positively, related to the physical impact of fatigue. The relationship between disease activity and fatigue varied relative to cortical complexity in the right superior temporal gyrus (ΔR^2^ = 0.062, *F* = 5.558, *p* = 0.023) and the right superior frontal gyrus (ΔR^2^ = 0.058, *F* = 4.059, *p* = 0.050).

**Discussion:**

The present findings expand our understanding of the complex brain-gut interactions linking disease activity and fatigue in Crohn’s disease relative to underlying differences in cortical complexity.

## 1 Introduction

Crohn’s disease is a form of inflammatory bowel disease (IBD) that is characterized by chronic, alternating periods of remitting and relapsing inflammation of the gastrointestinal tract resulting in symptoms of abdominal pain, diarrhea and weight loss (Singh and Bernstein, 2022). Fatigue is one of the most common extraintestinal symptoms in Crohn’s disease and affects persons during active disease and remission (Graff et al., 2011; D’Silva et al., 2022). Fatigue in Crohn’s disease has been designated as a top-rated research priority owing to its negative impact on health-related quality of life (Radford et al., 2021; Dibley et al., 2017). While various risk factors (including disease activity) have been identified (Villoria et al., 2017; Pal et al., 2024; Graff et al., 2013), the pathophysiology of fatigue in Crohn’s disease remains poorly understood. Recent research suggests that the physical and cognitive impacts of fatigue are related to resting state functional connectivity (FC), and that the relationship between disease activity and self-reported fatigue depends on underlying variability in resting state FC (McIver et al., 2025). Yet, the potential moderating effect of brain structure on the relationship between disease activity and self-reported fatigue has yet to be comparably examined. Characterizing fatigue-related differences in brain structure among persons with Crohn’s disease would address gaps in our understanding of the brain-gut axis mechanisms underlying such extraintestinal symptoms, and potentially inform novel therapeutic targets.

Structural and functional neural correlates of fatigue in Crohn’s disease have been identified in recent research. Studies using voxel-based morphometry (VBM) of magnetic resonance imaging (MRI) have yielded inconsistent results on the relationship between fatigue and grey matter volume. While Thapaliya et al. (2022) reported an inverse relationship between fatigue and grey matter volume in brain regions including the right supplementary motor area and white matter volume in the left cerebellum, Thomann et al. (2024) reported a positive association between fatigue and grey matter volume in the right middle temporal gyrus and cerebellum and left inferior temporal gyrus. In contrast to VBM, surfaced-based morphometry uses brain surface meshes for more accurate spatial registration than volume-based registration (Desai et al., 2005). Surface-based morphometry produces multiple metrics including cortical complexity. Cortical complexity offers an estimation of gyrification and reflects a combination of sulcal depth, the frequency of folding, and the convolution of gyral shape (Im et al., 2006). Cortical complexity is an understudied surface-based morphometry measure in Crohn’s disease, despite being linked to a variety of neurological disorders (Meregalli et al., 2022) and research that has identified differences in cortical complexity among persons with IBD in the absence of detectable grey matter volume differences (Kornelsen et al., 2023). Notably, in addition to volumetric differences, Thapaliya et al. (2022) reported that greater fatigue was related to decreased cortical thickness (another surface-based morphometry measure) in the right parahippocampal gyrus, frontal pole, left orbitofrontal cortex, temporal fusiform, inferior temporal, postcentral, and middle frontal gyri, highlighting a need to advance our understandings of structural differences in persons with Crohn’s disease by incorporating more surface-based measures.

While structural differences in persons with Crohn’s disease have been linked to fatigue, the self-reported impact of this symptom can be measured relative to separate physical, cognitive, and psychosocial domains of functioning (Marrie et al., 2024). A recent study from our group examined the association between these separately defined domains of self-reported fatigue impact and resting state FC among persons with Crohn’s disease. We found that FC was related to physical and cognitive self-reported impacts of fatigue (McIver et al., 2025). Notably, the separate domains for the impact of fatigue have yet to be studied relative to cortical complexity in Crohn’s disease. Further, while prior research has shown fatigue-related brain structure differences between persons with active disease compared to those in remission (Thomann et al., 2024), the implications of varied cortical complexity have yet to be studied relative to the link between disease activity and fatigue. We therefore aimed to (1) test for a relationship between cortical complexity and the self-reported impact of fatigue in the physical, cognitive, and psychosocial domains, and (2) determine whether the association between disease activity and self-reported fatigue varies relative to cortical complexity. With prior studies on fatigue-related differences in brain structure and function implicating diffuse involvement of temporal, frontal, and parietal brain regions, we hypothesize that the association between cortical complexity and the self-reported impact of physical, cognitive, and psychosocial fatigue may vary relative to the sensorimotor, cognitive, or social-affective processes typically linked to previously reported fatigue-related neural correlates. Further, we hypothesize that cortical complexity in brain regions associated with self-reported fatigue in the current study will exhibit a moderating effect on the relationship between disease activity and fatigue.

## 2 Materials and methods

### 2.1 Participants

The present research was conducted as part of a parent longitudinal comparative study investigating psychiatric comorbidity in immune-mediated inflammatory disease (Marrie et al., 2018). In the current sub-study, we examined the relationship between cortical complexity and the physical, cognitive, and psychosocial self-reported impacts of fatigue among 49 adult participants with Crohn’s disease and 49 healthy controls. Adults with Crohn’s disease were recruited through this parent study and healthy controls were recruited through a separate sub-study (Uddin et al., 2022). Studies were approved by the University of Manitoba Health Research Ethics Board and all participants provided informed consent (Marrie et al., 2018; Uddin et al., 2022). Inclusion criteria for all study participants included being ≥ 18 years of age, English-speaking, and screening clear of MRI contraindications, history of brain tumor, neurodegenerative disease, or immune-mediated inflammatory disease (other than Crohn’s disease for the patient group). In addition to the exclusion criteria for participants with Crohn’s disease, healthy controls were additionally screened for history of traumatic brain injury with loss of consciousness or amnesia, chronic medical conditions including hypertension, intellectual disability or cognitive impairment, psychiatric disorders on the Structured Clinical Interview for DSM-IV, and any chronic medication use other than contraceptives or hormone replacement therapy, as detailed previously (Marrie et al., 2018; Uddin et al., 2022). Only participants who underwent brain MRI were included in the sub-study, with groups formed such that age and ratio of women to men did not significantly differ between healthy controls and those with Crohn’s disease (Table 1).

**Table 1:** Demographics, disease metrics, self-report measures.

Variable label	CD (*n* = 49)	HC (*n* = 49)	Test statistic
Age, years	52.85 ± 13.85 (22–77)	47.56 ± 14.28 (19–81)	*t*(96) = 1.86, *p* = 0.066
Sex, women	35 (71%)	30 (61%)	χ2(1) = 1.142, *p* = 0.285
Active (HBI ≥ 5)/inactive disease	^a^16 (35%)/30(65%)		
CD location	20 L1 (41%); 5 L2 (10%); 24 L3 (49%); 1 L4 (2%)		
CD behavior	17 B1 (35%); 17 (35%) B2; 18 B3 (37%); 5 P (10%)		
CD disease duration (yrs)	27.69 ± 13.18 (6–57)		
CD medication use	5-ASA|5-ASA oral (*n* = 1|5), thiopurines (*n* = 13), methotrexate (*n* = 2), antibodies to TNF (*n* = 19), ustekinumab (*n* = 6), vedolizumab (*n* = 1)		
FIS physical	10.63 ± 10.01 (0–40)	2.84 ± 4.40 (0–18)	*t*(65.87) = 4.99, *p <* 0.001
FIS cognitive	11.18 ± 9.93 (0–38)	3.96 ± 4.99 (0–20)	*t*(70.76) = 4.55, *p <* 0.001
FIS psychosocial	18.57 ± 18.38 (0–71)	4.53 ± 6.58 (0–27)	*t*(60.10) = 5.04, *p <* 0.001
FIS total	40.39 ± 37.0 (0–149)	11.33 ± 14.99 (0–57)	*t*(63.35) = 5.10, *p <* 0.001

Age, HADS-D, and FIS (total and subscales) reported in mean ± standard deviation (range). Sex and disease activity reported by number of participants, with percentage of participants who were women and the percentage of those in active vs inactive disease state provided in brackets. HC = healthy controls, CD = Crohn’s disease, L1 = ileum, L2 = colon, L3 = small bowel/colon, L4 = upper GI which can accompany any of the L1, L2, or L3, B1 = inflammatory, B2 = fibrostenosing, B3 = fistula, P = perianal disease, HADS-D = Hospital Anxiety and Depression Scale- Depression subscale, FIS = Fatigue Impact Scale.

a Data are missing for three CD Harvey-Bradshaw scores. Fifteen participants with CD were not using any IBD medication.

### 2.2 Self-report and diagnostic measures

All participants completed the Fatigue Impact Scale (FIS) (Marrie et al., 2024; Fisk et al., 1994). This validated self-report measure consists of 40 items that assess the impact of fatigue across three domains: Cognitive functioning (10 items); Physical functioning (10 items); and Psychosocial functioning (20 items). Participants were asked to rate the extent to which fatigue has caused problems for them (0 = “no problem” to 4 = “extreme problem”) in relation to exemplar statements (e.g., “I am less able to complete tasks that require physical effort”). The depression subscale of the Hospital Anxiety and Depression Scale (HADS) (Zigmond and Snaith, 1983) was collected to confirm that participants were not affected by a clinically significant level of symptoms, with both group means falling below the clinically meaningful threshold of 11 (HC*M*_HADS-D_ = 1.22, *SD* = 1.56; CD*M*_HADS-D_ = 3.96, *SD* = 3.77). Disease duration as well as activity was noted among those with Crohn’s disease, with 16 participants scoring ≥5 (active range) on the Harvey Bradshaw Index (HBI) (Evertsz’ et al., 2013) and 30 participants scoring below (inactive range). Medication use as well as disease phenotyping for location and behavior (via the Montreal classification system) (Satsangi et al., 2006) were recorded (Table 1).

### 2.3 Neuroimaging data acquisition and pre-processing

Neuroimaging data were acquired using a 3 T Siemens TIM Trio MRI system (Siemens Healthcare, Erlangen, Germany), with acquisition parameters and preprocessing detailed in previous work from our group (Uddin et al., 2022). Briefly, high resolution anatomical, whole-brain T1-weighted (T1w) images were acquired using a 3D magnetization prepared rapid acquisition gradient-echo (MPRAGE) sequence with the following scan parameters: repetition time (TR) = 1,900 ms, echo time (TE) = 2.47 ms, inversion time (TI) = 900 ms, flip angle = 9°, GRAPPA = 2, matrix size = 256 × 256, field of view (FOV) = 250 × 250 mm^2^, number of slices = 176, slice thickness = 1.00 mm, number of averages = 1, bandwidth (BW) = 170 Hz/Px, echo spacing (ESP) = 7.3 ms, spatial resolution = 0.98 × 0.98 × 1.00 mm^3^, acquisition time (TA) = 4:26 min.

Preprocessing steps included skull-stripping, bias correction, segmentation, denoising, spatial normalization, modulation and smoothing. Data were segmented using default settings for spatial registration including shooting registration to the CAT12 MNI152NLin2009cAsym template for three voxel classes (grey matter, GM; white matter, WM and cerebral spinal fluid, CSF) and with the voxel size thickness estimation set at 0.5 mm for surfaces. Whole brain surface estimation was performed with affine and non-linear modulated normalized output.

### 2.4 Data analysis

Cortical complexity analyses were run using the default settings in the Computational Anatomy Toolbox 12 (CAT12, expert mode, version 12.8.1; Structural Brain Mapping Group, University of Jena, Germany) implemented in SPM12 (Statistical Parametric Mapping, Institute of Neurology, London, UK) (Gaser et al., 2022). The left and right hemisphere surface data were resampled and merged to a single 32 k mesh and smoothed with the recommended 20 mm FWHM kernel. Using a general linear model, the simple main effect fatigue (via FIS scores) was tested within Crohn’s disease and separately within healthy controls, while controlling for age. Separate analyses were run for each of the subscales of the FIS (physical, cognitive, and psychosocial). Between-group contrasts compared the relationship between cortical complexity and FIS scores between the groups (healthy controls > Crohn’s disease; healthy controls < Crohn’s disease). We employed an extent threshold to correct for multiple comparisons, following the instructions given in the CAT12 manual. This is applied in a two-step process, where first the *p*-value is applied to the statistical map for all voxels and the voxels significant at *p* < 0.001 are displayed. The extent threshold is also shown for the resulting statistical map, which is an empirical value representing the expected number of vertices per cluster. In the second step, the results are displayed again with both the *p* < 0.001 and the extent threshold value applied, which then displays only the clusters that met both criteria. Results were displayed at *p* < 0.001 with the relevant empirically determined extent threshold (*k*) applied. Significant clusters were labeled with the Desikan-Killiany DK40 atlas (Desikan et al., 2006). Post-hoc analyses were performed using the PROCESS macro (Hayes, 2018) in SPSS and tested for a moderating effect of cortical complexity on the positive relationship between disease activity (via scores on the HBI) and fatigue (via total scores on the FIS). Given that scores on the HBI were missing for three of the participants with CD, the moderation analyses were completed including the data from only those 46 participants from whom HBI data was complete. Four moderation analyses were performed, with the moderator in each model being the cortical complexity within one of the significant clusters identified in main effect of fatigue on cortical complexity analyses in the Crohn’s disease group: Model 1: right superior temporal gyrus; Model 2: right superior frontal gyrus; Model 3: right supramarginal gyrus; Model 4: left superior frontal gyrus. Using an ordinary least squares path analysis approach, conditional effects were calculated to perform simple slope analyses at low (16th percentile), medium (50th percentile), and high (84th percentile) levels. These analyses included a bias correction with 5,000 bootstrapped samples and confidence intervals of 95%.

## 3 Results

**Figure 1:**
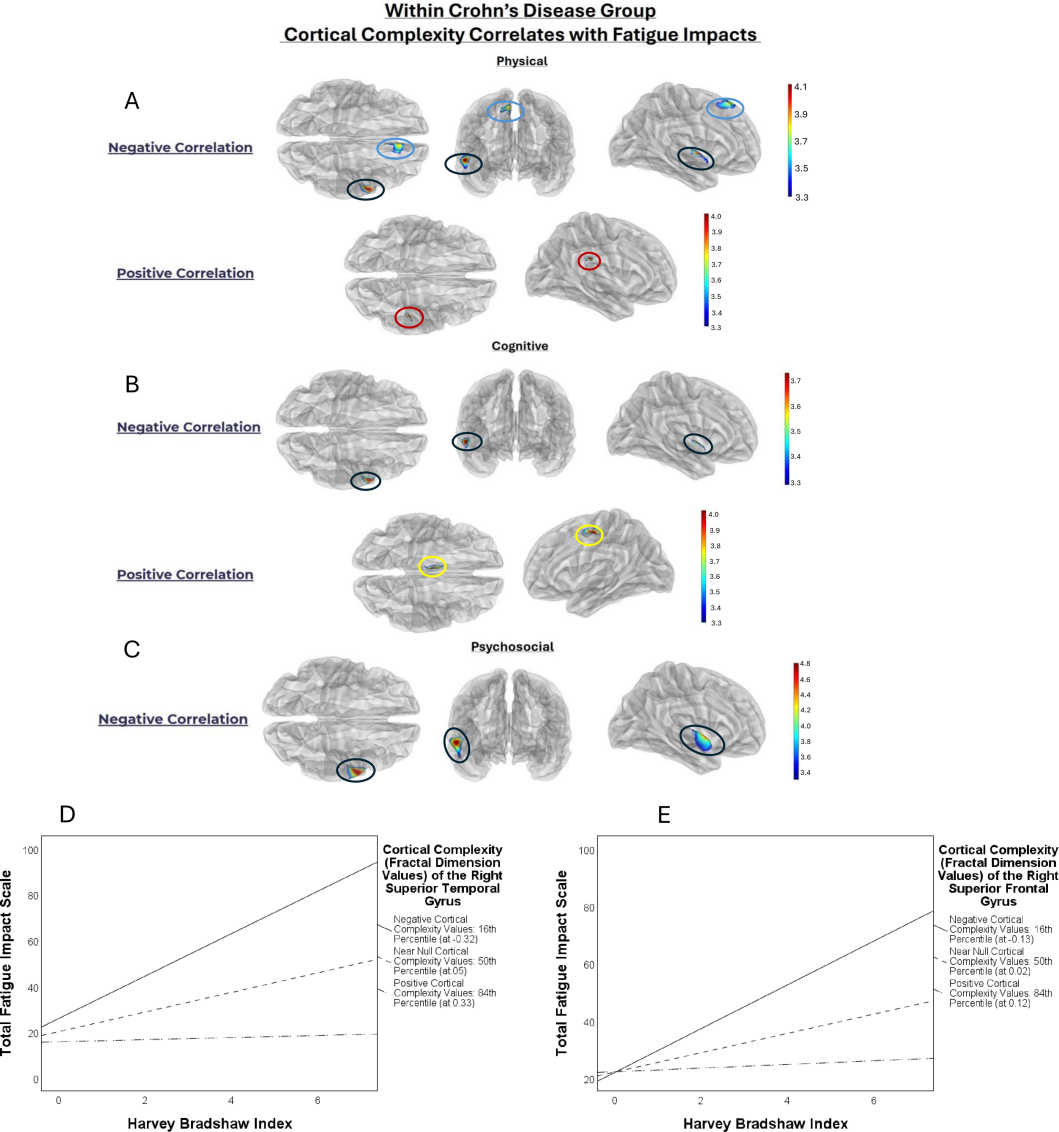
Simple main effect of fatigue within physical, cognitive, and psychosocial domains on cortical complexity in the Crohn’s disease group. **(A–C)** Show semi-transparent glass-brain renderings presenting the location and significance of the association between cortical complexity and the impact of fatigue among persons with CD, with corresponding color-bar to the right indicating *t*-values for color mapping significance. The *t*-values of the colour bar correspond to the test statistic from the GLMs for the with-group main-effect of fatigue. In **(A)**, results are presented for the relationship between cortical complexity and the physical domain of fatigue for the Crohn’s disease groups, with the significant cluster in the right superior temporal gyrus circled in black, and the right superior frontal gyrus circled in blue. Views progress from the top (axial), front (coronal), and right (sagittal) from left to right. Shown below in views from the top and right is significant cluster in the right supramarginal gyrus circled in red. **(B)** Shows the location and significance of the association between cortical complexity and the cognitive impact of fatigue among persons with CD. A cluster in the right superior temporal gyrus is circled in black and a cluster in the left superior frontal gyrus is circled in yellow. **(C)** Shows the location and significance of the association between cortical complexity and the psychosocial impact of fatigue among persons with CD. As with the physical and cognitive impacts of fatigue, a cluster in the right superior temporal gyrus is circled in black. **(D)** and **(E)** show line graphs visualizing the moderating effect of cortical complexity from the right superior temporal gyrus and right superior frontal gyrus, respectively.

**Figure 2:**
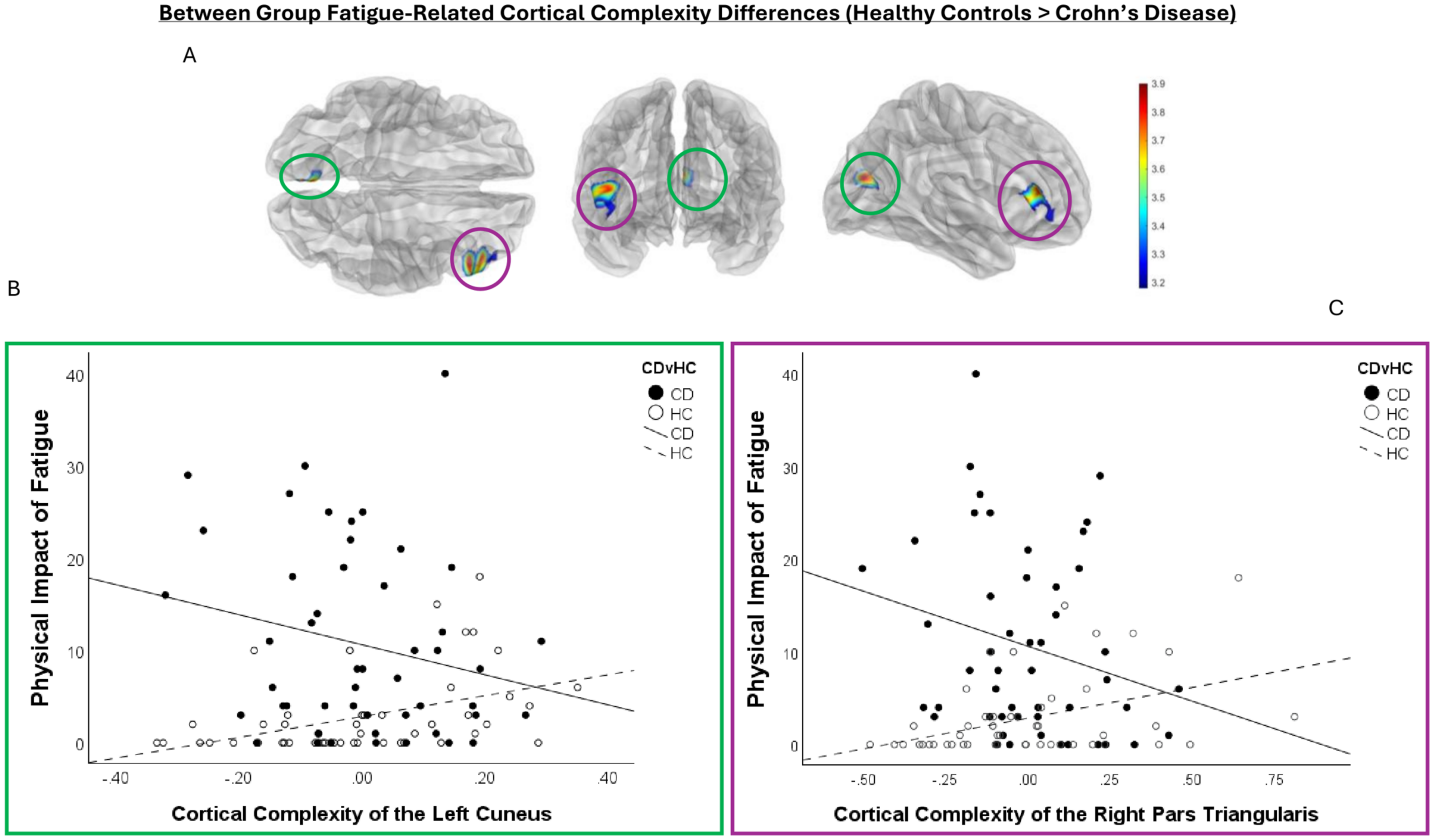
Results of the between group contrast HC>CD showing differences between groups in the association between cortical complexity and fatigue. **(A)** Shows semi-transparent glass-brain renderings presenting the location and significance of the difference between HC and CD in the association between cortical complexity and the impact of fatigue, with corresponding color-bar to the right indicating *t*-values for color mapping significance. The *t*-values of the colour bar correspond to the test statistic from the between-group contrast. Significant clusters were identified in the right pars triangularis (circled in purple) and the left cuneus (circled in green). For HC, cortical complexity in these clusters is higher among persons who exhibit greater physical impact of fatigue. The CD group exhibited the opposite effect, with greater physical impact of fatigue being associated with lower cortical complexity in these clusters. **(B)** Shows a scatterplot corresponding to the significant cluster in the left cuneus. The scatterplot for the right pars triangularis is presented in **(C)**.

The Crohn’s disease group reported significantly higher fatigue than healthy controls (Table 1). In the Crohn’s disease group, higher fatigue across all domains was associated with lower cortical complexity in the right superior temporal gyrus (Figure 1; Table 2). Higher physical fatigue was associated with lower cortical complexity in the right superior frontal gyrus and greater cortical complexity in the right supramarginal gyrus. Higher cognitive fatigue was associated with greater cortical complexity in the left superior frontal gyrus (Figure 1; Table 2). The healthy controls > Crohn’s disease contrast revealed greater fatigue-related cortical complexity for regions including the left cuneus/peri-calcarine gyrus and the right pars triangularis (Figure 2). There were no significant results from the healthy controls < Crohn’s disease contrast. Results of the post-hoc moderation analyses identified a moderating effect for the cortical complexity within the right superior temporal gyrus (ΔR^2^ = 0.062, *F* = 5.558, *p* = 0.023) and the right superior frontal gyrus (ΔR^2^ = 0.058, *F* = 4.059, *p* = 0.050) on the relationship between disease activity and the total impact of fatigue. Greater disease activity was significantly, positively, associated with total impact of fatigue when cortical complexity values in the right superior temporal gyrus were lowest, at both the 16th (*b* = 9.257, *SE* = 2.062, *t* = 4.490, *LLCI* = 5.096 *ULCI* = 13.417, *p* = 0.0001), and 50th percentile (*b* = 4.278, *SE* = 1.390, *t* = 3.078, *LLCI* = 1.473 *ULCI* = 7.083, *p* = 0.004). When cortical complexity values were highest (at the 84th percentile), total impact of fatigue was not related to disease activity (*b* = 0.4532, *SE* = 2.4917, *t* = 0.1819, *LLCI* = −4.5753 *ULCI* = 5.4817, *p* = 0.8565).

**Table 2:** Within- and between- group cortical complexity associations with the impact of fatigue across each domain (physical, cognitive, and psychosocial).

Group	Significant clusters	Test statistic	Cluster size (k)	*p* value
CD				
Physical	Right superior frontal gyrus	*t* = 3.8	100	*p* < 0.001
Negative	Right superior temporal gyrus	*t* = 4.1	79	*p* < 0.001
Positive	Right supramarginal gyrus	*t* = 4.0	57	*p* < 0.001
Cognitive				
Negative	Right superior temporal gyrus	*t* = 3.7	43	*p* < 0.001
Positive	Left superior frontal gyrus extending into paracentral	*t* = 4.0	71	*p* < 0.001
Psychosocial				
Negative	Right superior temporal gyrus	*t* = 4.8	204	*p* < 0.001
HC				
Physical				
Positive	Right pars triangularis extending into the rostral Middle Frontal Gyrus	*t* = 3.6	45	*p* < 0.001
	Left superior temporal gyrus extending into the supramarginal gyrus	*t* = 4.1	88	*p* < 0.001
HC>CD				
Physical	Right pars triangularis extending into rostral middle frontal gyrus and pars orbitalis	*t* = 3.9	286	*p* < 0.001
	Left cuneus extending into peri-calcarine	*t* = 3.9	65	*p* < 0.001

Similarly, greater total impact of fatigue was only associated with greater disease activity when cortical complexity in the right superior frontal gyrus was lowest and negative at the 16th percentile (*b* = 7.624, *SE* = 1.861, *t* = 4.106, *LLCI* = 3.886 *ULCI* = 11.398, *p* = 0.0002). There was no significant relationship between disease activity and fatigue impact when cortical complexity was positive at the 50th (*b* = 3.370, *SE* = 1.908, *t* = 1.767, *LLCI* = −0.480 *ULCI* = 7.219, *p* = 0.085) and 84th percentiles (*b* = 0.639, *SE* = 2.909, *t* = 0.220, *LLCI* = −5.232 *ULCI* = 6.510, *p* = 0.827). Corresponding values for the cortical complexity (fractal dimensions) are shown in panel D of Figure 1. Models 3 and 4 did not identify any significant moderating effects for cortical complexity in the remaining significant clusters.

## 4 Discussion

The pathophysiology of fatigue in Crohn’s disease can be better understood by examining cortical-level correlates of the symptom, highlighting differences in the brain-gut axis that play a role in extraintestinal symptom manifestation. While decreased cortical complexity has been reported for a range of neurological disorders (Meregalli et al., 2022), scant research has examined this surface-based morphometry metric in the context of Crohn’s disease. Further, the research examining neural correlates of fatigue in Crohn’s disease has rarely examined the impact of fatigue across different domains of functioning. In the present study, lower values of temporal and frontal cortical complexity were observed among persons with Crohn’s disease who reported more severe impact of fatigue, with varied relationships observed relative to the physical, cognitive, and psychosocial impacts of fatigue. While persons in active disease have previously been reported to exhibit distinct fatigue-related correlates compared to those in remission (Thomann et al., 2024), the present study strengthens our understanding of the relationship between disease activity and fatigue by demonstrating the moderating effect of complexity on that relationship.

Higher self-reported impact of fatigue across each domain was associated with lower values of cortical complexity in the right superior temporal gyrus among persons with Crohn’s disease. Increased responding of the superior temporal gyrus has previously been implicated in experimental induction of cognitive fatigue (i.e., acute self-reported fatigue responses following an experimental task) in persons with Chronic Fatigue Syndrome compared to healthy individuals (Cook et al., 2007). Further, a longitudinal neuroimaging study of fatigue in multiple sclerosis (which, like Crohn’s disease, is an immune-mediated inflammatory disease), found that the right temporal cortex showed fatigue-related decreases in fractional anisotropy, suggesting a link to diminished white matter integrity (Yarraguntla et al., 2018). The present findings suggest that fatigue-related structural differences in the right superior temporal gyrus are part of the pathology of persistent/trait-level, self-reported fatigue in Crohn’s disease as well.

Cognitive and physical fatigue are differently related to cortical complexity of the superior frontal gyrus. Cortical complexity values in the left superior frontal gyrus were higher among persons with Crohn’s disease who reported a greater cognitive impact of fatigue. In contrast, cortical complexity values of the right superior frontal gyrus were lower among persons with Crohn’s disease who reported greater physical impact of fatigue. While the left superior frontal gyrus is implicated in higher cognitive functioning and working memory (Du Boisgueheneuc et al., 2006), the right superior frontal gyrus plays a role in impulse response control and motor urgency (Hu et al., 2016). Persons with IBD have previously been found to exhibit deficits in executive function (working memory in particular) (Hopkins et al., 2021). Given that cortical complexity changes have been linked to learning (Im et al., 2006; Meregalli et al., 2022), future research should assess whether increased cortical complexity of the left superior frontal gyrus may reflect compensatory structural changes related to potential differences in executive functioning.

Increased cortical complexity of the right supramarginal gyrus is also related to higher self-reported impact of physical fatigue. Hyperactivity of the right supramarginal gyrus has been linked to motor impairment after subcortical ischemic stroke (Zhang, 2025). Incorporating assessments of physical and cognitive performance were beyond the scope of the current study, raising a potential limitation. Nonetheless, prior research has identified decreased complex psychomotoric reaction times in persons with IBD (Tadin Hadjina et al., 2019), and fatigue has been linked with decreased physical activity and fitness (including muscle strength) in persons with IBD (Vogelaar et al., 2015; Demers et al., 2025). Future functional neuroimaging work could employ motor task-based paradigms to elucidate whether fatigue-related cortical complexity in the right superior frontal and supramarginal gyri is associated with deficits in motor functioning and determine if this may be related to increased physical fatigue among persons with Crohn’s disease. The physical impact of fatigue is also the only domain in which healthy controls exhibited an association with cortical complexity, albeit with limited variability in fatigue scores and a low group mean warranting cautious interpretation. The regions identified are nonetheless involved in imagining sensations of fatigue (Tomasino et al., 2022), likely reflecting a role for these regions in normative processing of fatigue perception.

Results of the moderation analyses shed light on the link between disease activity and the impact of fatigue, with the strongest association being observed among persons with Crohn’s disease who exhibited the lowest cortical complexity values in the right superior temporal and frontal gyri. Other studies have either linked fatigue to disease activity (Gibble et al., 2024) or compared the fatigue-related structural differences between groups of persons in active disease to those in remission (Thomann et al., 2024). The current findings build on that research by providing context for how the relationship between disease activity and fatigue depends on underlying brain structure variability. In prior work from our lab, we observed a similar moderating effect for FC involving the temporal lobe (McIver et al., 2025). The current study helps to characterize cortical-level differences in the brain-gut axis that are implicated in the connection between enteric pathology (via increased disease activity) and perception of extraintestinal symptoms such as fatigue. To build upon the present contextualization of the interaction between disease activity, fatigue, and brain structure, future research should employ mediation analyses to test whether variability in cortical structure or functioning operate as a mechanism through which disease activity influences fatigue in Crohn’s disease. The brain-gut axis is a bidirectional communication system, with the gut influencing the brain in the production and expression of neurotransmitters, modulation of sensory nerves, and mucosal immune regulation and the brain influencing the gut in the alteration of motility, intestinal permeability, and immune function (Carabotti et al., 2015). As such, further research will be necessary to determine whether decreases in cortical complexity precede or follow the progression of disease activity and the impact it has on fatigue. With decreases in cortical complexity recently being identified as a potential predictor of disability among persons with other immune mediated inflammatory diseases like MS (Roura et al., 2021), the present findings suggest that decreased cortical complexity in relation to greater fatigue should be further explored as a risk factor for compromised cognitive and physical performance among persons with Crohn’s disease.

### 4.1 Limitations and future directions

The cross-sectional design of the current study raises a limitation to inferences we can make regarding causality. Longitudinal research on brain structure and function changes across time in Crohn’s disease is sorely lacking and is an essential next step toward understanding the mechanistic interaction between brain structure and the association between disease activity and fatigue. The current results are specific to self-reported measures of fatigue, the HBI as a measure of disease activity, and cortical complexity as a composite surface-based measure of morphometry. As such, future works can provide a more comprehensive understanding of the relationship between fatigue, disease activity, and brain structure by incorporating physical measures of fatigue, physiological markers for inflammation like C-reactive protein or fecal calprotectin, and additional neuroimaging measures such as cerebral profusion and metabolism. An added intricacy of studying neural mechanisms involved in Crohn’s disease is the varied use of medications across participants. The current study design does not facilitate comparison of neural correlates underlying fatigue relative to the different medications in use. It is possible that varied medication use may contribute to a presently unstudied variability in cortical complexity and its relation to fatigue. An interesting area for future research could be to explore whether different medications impact either the acutely malleable neural correlates such as resting state FC or the more stable metrics such as differences in brain structure, and how any changes observed for persons on varied medications relates to their experience of symptoms such as fatigue.

A further potential limitation is that the present findings are limited to persons with Crohn’s disease, necessitating further research among persons with ulcerative colitis to determine whether these findings are generalizable across different forms of IBD. Given that neither the Crohn’s disease group nor the healthy controls exhibited scores for depressive symptoms that met the criteria for clinical significance in the current study, future research may aim to compare fatigue-related morphology or function between persons with Crohn’s disease who exhibit depression compared to those who do not, in order to disentangle any potential overlapping neural correlates. In addition, while fatigue has been found to be a more prominent symptom among women with IBD than among men (Kamp et al., 2025; Blumenstein and Sonnenberg, 2023), there were no differences between the women and men in the current participant sample (Supplementary Table 1) and the present study did not explore interactions between fatigue, cortical complexity, and sex or gender. Supplementary Table 2 reports results for both within- and between-group cortical complexity contrasts while statistically controlling for age and sex, revealing much consistency with our initial analyses that relied upon balancing these variables between groups. While sample sizes in the current study were sufficient for the presently conducted analyses, future research with larger sample sizes could be used to focus on generating subgroups to support, for example, testing for sex-based interactions or direct contrasting of groups of persons in remission to those in active disease state. Consideration of such subgroups would provide a more nuanced understanding of the neural mechanisms that contribute to symptom experiences for persons with Crohn’s disease. It is possible that with larger subgroups of men and women within the Crohn’s disease group, differences may emerge between men and women that may influence the relationship between trait-level fatigue and cortical complexity. Similarly, sex or gender differences may be more pronounced when fatigue is more extreme. With the current findings demonstrating the relationship between trait-level, self-reported fatigue and cortical complexity in Crohn’s disease in a mixed sample, future studies can be designed to explore whether men and women with Crohn’s disease differ in the relationship between fatigue and cortical complexity.

In conclusion, for persons with Crohn’s disease, lower cortical complexity values in the right superior temporal gyrus are associated with increased impact of cognitive, physical, and psychosocial fatigue, implicating decreased cortical complexity of this brain region in the overall experience of fatigue. Meanwhile, the cortical complexity of the superior frontal gyrus relates to the impact of fatigue differently within the cognitive domain compared to the physical. The physical impact of fatigue is further tied to higher values of cortical complexity in the right supramarginal gyrus, likely relating to the previously reported altered relationship between physical fatigue and functional connectivity of this region in persons with Crohn’s disease (McIver et al., 2025), and reinforcing the importance of this brain region specifically in the physical domain. Lower cortical complexity within the right superior temporal and frontal gyri appears to play a role in defining the relationship between disease activity and fatigue in persons with Crohn’s disease, highlighting cortical-level differences along the brain-gut axis that contribute to extra intestinal symptoms such as fatigue in persons with Crohn’s disease. Underlying variability in cortical complexity of brain regions involved in executive functioning and motor control may contribute to the likelihood of experiencing fatigue in relation to disease activity.

## Data Availability

The datasets presented in this article are not readily available because the data underlying this article cannot be shared publicly due to the authors not having permission to share study materials including data. Requests to access the datasets should be directed to Jennifer.Kornelsen@umanitoba.ca.
