# Supplementary material for: Physical, cognitive, and psychosocial fatigue are differently related to cortical complexity of superior temporal and frontal brain regions in Crohn’s disease
Source: Front Neuroimaging. 2026 Apr 16;5:1814006. doi: 10.3389/fnimg.2026.1814006 (PMC13128373; doi:10.3389/fnimg.2026.1814006)
Supplement: Supplementary file 1 [file Table_1.docx]

Supplementary Material

# Supplementary Tables

For more information on Supplementary Material and for details on the different file types accepted, please see [here](https://www.frontiersin.org/guidelines/author-guidelines#supplementary-material).

**Table 1. Fatigue Impact Scale Scores for Men and Women, Separated by Group; Crohn’s Disease Group and Healthy Control Group**

|  | **CD Women (n=35)** | **CD Men (n = 14)** | **Test Statistic** |
| --- | --- | --- | --- |
| FIS Physical | 11.46±10.92 | 8.57±7.15 | *t*(47)=0.91, *p*=0.184 |
| FIS Cognitive | 12.23±10.68 | 8.57±7.54 | *t*(47)=1.17, *p*=0.124 |
| FIS Psychosocial | 20.29±20.10 | 14.29±12.77 | *t*(47)=1.03, *p*=0.001 |
|  | **HC Women (n=30)** | **HC Men (n = 19)** | **Test Statistic** |
| FIS Physical | 2.57±4.16 | 3.26±4.84 | *t*(47)=0.54, *p*=0.297 |
| FIS Cognitive | 3.27±3.87 | 5.05±6.34 | *t*(26.58)=1.11, *p*=0.140 |
| FIS Psychosocial | 3.80±4.57 | 5.68±8.91 | *t*(24.07)=0.85, *p*=0.201 |
| FIS Total | 9.63±11.25 | 14.00±19.57 | *t*(25.62)=0.884, *p*=0.192 |

FIS = Fatigue Impact Scale.

**Table 2. Within- and Between- Group Cortical Complexity associations with the Impact of Fatigue Across Each Domain (Physical, Cognitive, and Psychosocial) *CONTROLLING FOR AGE & SEX, (AS WELL AS DISEASE DURATION FOR CD WITHIN-GROUP)***

| **Group** | **Significant Clusters** | **Test Statistic** | **Cluster size (k)** | ***p* value** |
| --- | --- | --- | --- | --- |
| **CD** |  |  |  |  |
| **Physical** | right Superior Frontal Gyrus | *t*=3.7 | 60 | *p*<0.001 |
| ***Negative*** | right Superior Temporal Gyrus | *t*=4.1 | 71 | *p*<0.001 |
|  | right Medial Orbtiofrontal Gyrus | *t*=3.7 | 48 | *p*<0.001 |
|  |  |  |  |  |
| ***Positive*** | right Supramarginal Gyrus | *t*=3.8 | 40 | *p*<0.001 |
| **Cognitive** |  |  |  |  |
| ***Negative*** | right Superior Temporal Gyrus | *t*=3.7 | 42 | *p*<0.001 |
| ***Positive*** |  | *t*=3.8 | 43 | *p*<0.001 |
|  | left Superior Frontal  Gyrus extending into Paracentral |  |  |  |
| **Psychosocial** |  |  |  |  |
| ***Negative*** | right Superior Temporal Gyrus | *t*=4.7 | 197 | *p*<0.001 |
| **HC** |  |  |  |  |
| **Physical** |  |  |  |  |
| ***Positive*** | right rostral Middle Frontal Gyrus extending into the Pars Triangularis | *t*=3.7 | 55 | *p*<0.001 |
|  | left Superior Temporal Gyrus extending into the Supramarginal Gyrus | *t*=4.0 | 78 | *p*<0.001 |
| **HC>CD** |  |  |  |  |
| **Physical** | right Pars Triangularis extending into rostral Middle Frontal Gyrus and Pars Orbitalis | *t*=3.8 | 293 | *p*<0.001 |
|  | left Cuneus extending into Peri-calcarine | *t*=3.8 | 63 | *p*<0.001 |
